# Electrical storms induced by multiple shocks in catecholaminergic polymorphic ventricular tachycardia, spotlight

**DOI:** 10.1002/joa3.13122

**Published:** 2024-07-22

**Authors:** Hisaaki Aoki, Yoshihide Nakamura

**Affiliations:** ^1^ Department of Pediatric Cardiology Osaka Women's and Children's Hospital Osaka Japan

**Keywords:** catecholaminergic polymorphic ventricular tachycardia, electrical storm, implantable cardioverter defibrillator

## Abstract

An electrical storm ensued following multiple shocks by an implantable cardioverter defibrillator (ICD). The prevention of such electrical storms is prognostically important and includes revision of medical therapy, modification of ICD settings, cardiac sympathectomy, and ablations.

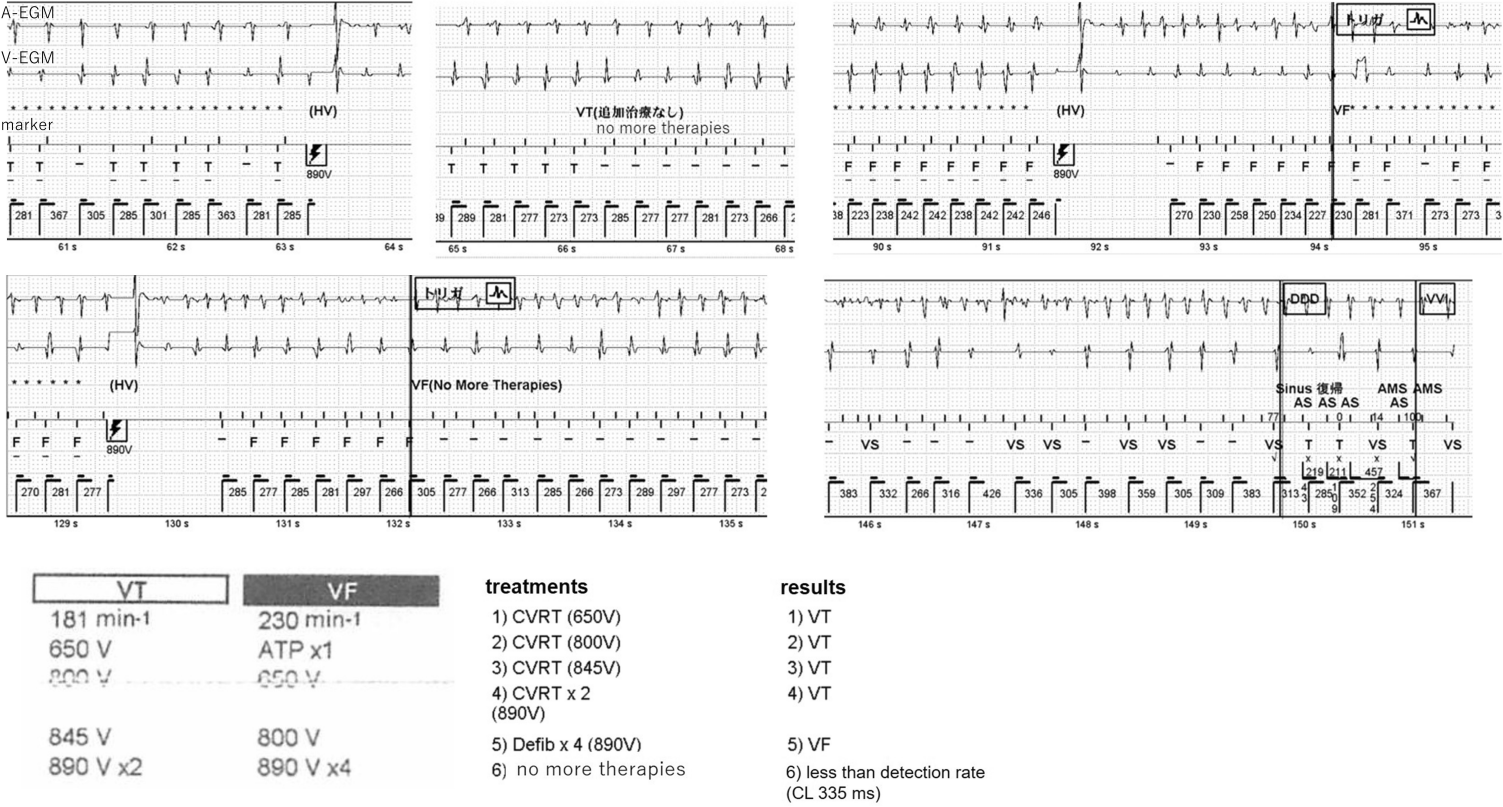

Implantable cardioverter defibrillator (ICD) therapy is controversial because of reported cases of ICD shocks causing arrhythmias and death in patients with catecholaminergic polymorphic ventricular tachycardia (CPVT).^1^ Recently, an international multicenter cohort study of ICD in CPVT was reported.^2^ During a median follow‐up of 8.0 years, seven patients (3.0%) experienced sudden cardiac death (SCD), all of whom did not have an ICD at the time of SCD. However, many arrhythmias recover spontaneously and it is important to avoid unnecessary ICD shocks. We report a case of recurrent electrical storm after ICD implantation in a patient with CPVT to highlight the importance of an optimal medical therapy including ICD settings.

The case is a 19‐year‐old man. He fainted while being chased during a bean‐throwing ceremony in kindergarten. He then experienced a syncopal episode while climbing a pole in elementary school. During an examination at his local doctor's office, he developed ventricular fibrillation (VF) and was brought to our hospital. The resting 12‐lead electrocardiography (ECG) yielded normal results; however, the exercise ECG demonstrated bidirectional ventricular tachycardia (VT). Genetic testing did not identify any RyR2 mutations. Following diagnosis with CPVT, the patient was started on propranolol. Subsequently, the patient experienced several episodes of syncope. At the age of 18 years old, he experienced a cardiopulmonary arrest during a fireworks incident and was admitted to our hospital. The patient did not ingest any medication on that particular day. After the admission, he continued to experience episodes of polymorphic VT and VF. Treatment involving propranolol and flecainide, followed by the implantation of ICD (Fortify DR, St. Jude Medical, Minnetonka, MN) for secondary prevention. Holter ECG before ICD implantation showed a maximal heart rate of 114 beats per minute (bpm). The treatment settings were configured with a two‐zone setup where the VT zone was set at 181 bpm, 24 intervals and the VF zone at 230 bpm, 12 intervals, and supraventricular tachycardia discrimination. He received multiple shocks at 1, 3, 5, and 6 months after implantation while traveling or riding a bicycle. The patient initially presented with VT and received five shocks. Subsequently, VT evolved to VF coexisting with supraVT. After four shocks, the arrhythmias spontaneously reverted to sinus rhythm (Figures 1 and 2). After increasing the flecainide dose (from 207 ng/mL before to 574 ng/mL after within the therapeutic range of 400–900 ng/mL), the ICD remained in active.

**FIGURE 1:**
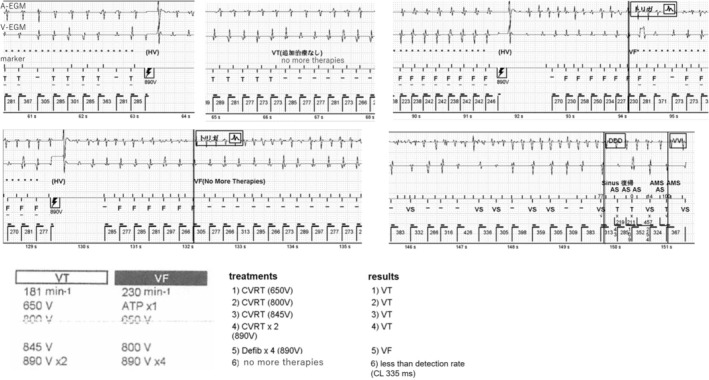
Episode 1, an electrical storm ensued following multiple shocks by an implantable cardioverter defibrillator (ICD). The ICD settings are configured with a two‐zone setup, comprising a ventricular tachycardia (VT) zone set at 181 beats per minute (bpm) with 24 intervals and a ventricular fibrillation (VF) zone set at 230 bpm with 12 intervals. Five shocks delivered to the patient initially diagnosed with VT. The VT then transitioned to VF coexisting with supraventricular tachycardia. After the delivery of four shocks, the arrhythmias spontaneously returned to sinus rhythm. 650 V, 650 volts; A‐EGM, atrial electrogram; ATP, anti‐tachycardia pacing; CVRT, conversion therapy; Defib, defibrillation; event markers such as T, F, VS, A, and AMS indicate VT event, VF event, ventricular sensed event, atrial‐sensed event, and auto mode switch, respectively; HV, high voltage therapy; V‐EGM, ventricular electrogram; VF, ventricular fibrillation; VT, ventricular tachycardia.

**FIGURE 2:**
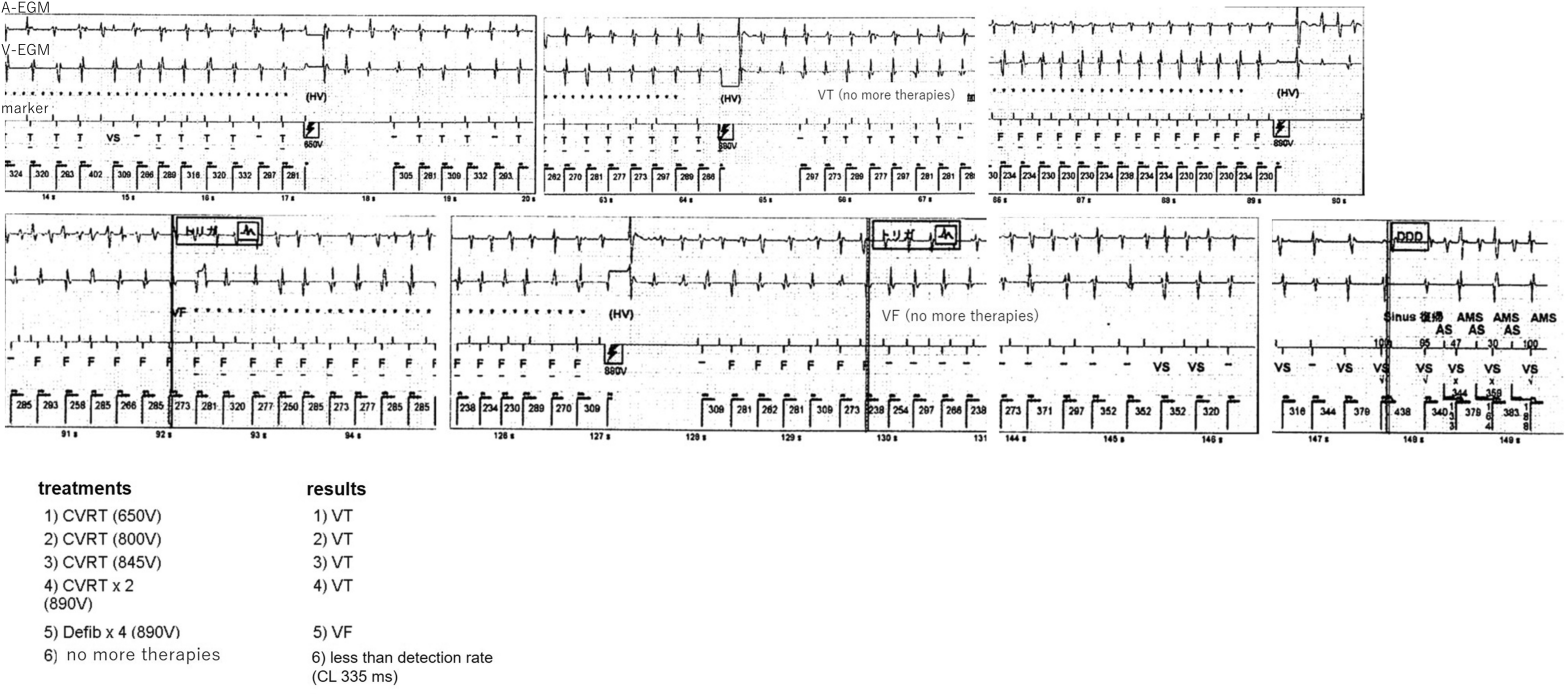
Episode 2, electrical storm same as Figure 1.

Although patients with CPVT have been reported to have high rates of appropriate shocks and to be saved by ICD shocks, ICD therapy remains controversial due to reported instances where ICD shocks have triggered arrhythmias and led to fatalities in patients with CPVT.^1^ The incidences of inappropriate shocks and complications reported were also high.^2^ Electrical storms occur in 6%–19% of the patients.^2^ In the present case, the patient experienced frequent ICD shocks for atrial tachycardia and VT. After the last shock, the patient spontaneously returned to sinus rhythm. Prevention of such electrical storms is prognostically important and includes revision of medical therapy, modification of ICD settings,^3^ cardiac sympathectomy, and ablations. Nadolol has been reported to be effective and should be considered when other beta‐blockers are used. Moreover maintaining adequate flecainide levels in the blood levels, increases detection rates, and prolongs detection interval.^3^ To mitigate the risk of shocks secondary to ventricular ectopy and nonsustained short runs of polymorphic VT after VT/VF termination, configuring a single VF zone to a substantially shorter cycle length (range, 200–260 ms) would have shortened the redetection intervals. Coupled with an increasing redetection duration, this approach may have effectively restricted shocks to sustained VF episodes. In the present case, the ventricular cycle length at the onset of VT was approximately 300 ms. After the shock, the VT was regular and accelerated to a cycle length of 230 ms. Subsequently, atrial tachycardia and atrial fibrillation ensued. To avoid ICD shock during VT, configuring ICD settings with either a single zone VF range of 200–250 ms or a two‐zone setup with VF zone 200 ms and a VT zone at 300 ms may have been acceptable. For an atrial lead, the two‐zone setting may delay the timing of the ICD shock and reduce storms. Subcutaneous ICD for CPVT is controversial due to an upper limit of 250 ms for the detection rate. Moreover, the setting does not allow for a prolonged detection period. The efficacy of cardiac sympathectomy has been reported in many cases and may reduce appropriate and inappropriate shocks after ICD implantation.^4^ Although it is not an indication for hereditary arrhythmia insurance in Japan, it should be considered particularly in patients with electrical storm or poor medication adherence.^5^ Catheter ablation of the bidirectional ventricular premature beats that trigger VF may become an adjunctive therapy in patients with refractory CPVT.^6^ In addition, cases of focal atrial tachycardia and atrial fibrillation have been reported.^7^ Given the lack of data in this area, the current HRS/EHRA/APHRS consensus document does not provide advice regarding catheter ablation in CPVT, but it seems likely that ablation will be part of the adjunctive therapy for these patients in the future.^8^

In CPVT, it is important to control both atrial and lethal ventricular arrhythmias. Multidisciplinary management should prevent the occurrence of arrhythmias.

## FUNDING INFORMATION

This research did not receive any specific grant from funding agencies in the public, commercial, or not‐for‐profit sectors.

## CONFLICT OF INTEREST STATEMENT

All of the authors have no conflicts to disclose.

## ETHICS STATEMENT

N/A.

## INFORMED CONSENT

The consent of the publication was obtained.
